# Early alterations of the innate and adaptive immune statuses in sepsis according to the type of underlying infection

**DOI:** 10.1186/cc9031

**Published:** 2010-05-26

**Authors:** Charalambos Gogos, Antigone Kotsaki, Aimilia Pelekanou, George Giannikopoulos, Ilia Vaki, Panagiota Maravitsa, Stephanos Adamis, Zoi Alexiou, George Andrianopoulos, Anastasia Antonopoulou, Sofia Athanassia, Fotini Baziaka, Aikaterini Charalambous, Sofia Christodoulou, Ioanna Dimopoulou, Ioannis Floros, Efthymia Giannitsioti, Panagiotis Gkanas, Aikaterini Ioakeimidou, Kyriaki Kanellakopoulou, Niki Karabela, Vassiliki Karagianni, Ioannis Katsarolis, Georgia Kontopithari, Petros Kopterides, Ioannis Koutelidakis, Pantelis Koutoukas, Hariklia Kranidioti, Michalis Lignos, Konstantinos Louis, Korina Lymberopoulou, Efstratios Mainas, Androniki Marioli, Charalambos Massouras, Irini Mavrou, Margarita Mpalla, Martha Michalia, Heleni Mylona, Vassilios Mytas, Ilias Papanikolaou, Konstantinos Papanikolaou, Maria Patrani, Ioannis Perdios, Diamantis Plachouras, Aikaterini Pistiki, Konstantinos Protopapas, Kalliopi Rigaki, Vissaria Sakka, Monika Sartzi, Vassilios Skouras, Maria Souli, Aikaterini Spyridaki, Ioannis Strouvalis, Thomas Tsaganos, George Zografos, Konstantinos Mandragos, Phylis Klouva-Molyvdas, Nina Maggina, Helen Giamarellou, Apostolos Armaganidis, Evangelos J Giamarellos-Bourboulis

**Affiliations:** 1 1st Department of Internal Medicine, University of Patras, Medical School, 26504 Rio, Greece; 2 4th Department of Internal Medicine, University of Athens, Medical School, ATTIKON General Hospital, 1 Rimini Str., 12462 Athens, Greece; 3 Department of Internal Medicine, Chios General Hospital, 2 Elena Venizelou Str., 82100 Chios, Greece; 4 2nd Department of Urology, "Sismanogleion" Athens Hospital, 1 Sismanogleiou Str., 15126 Maroussi, Greece; 5 1st Department of Internal Medicine, "Thriasion" Elefsina General Hospital, Leoforos Gennimata, 19600 Magoula, Greece; 6 Department of Internal Medicine, Argos General Hospital, 191 Korinthou Str., Argos, Greece; 7 Intensive Care Unit, "Ippokrateion" Athens General Hospital, 114 Vassilis Sofias Str., 11527 Athens, Greece; 8 1st Department of Internal Medicine, "G. Gennimatas" Athens Hospital, 154 Mesogeion Str., 11527 Athens, Greece; 9 2nd Department of Critical Care, University of Athens, Medical School, ATTIKON General Hospital, 1 Rimini Str., 12462 Athens, Greece; 10 Intensive Care Unit, "Laikon" Athens General Hospital, 17 Aghiou Thoma Str., 11527 Athens, Greece; 11 Department of Surgery, Nafplion General Hospital, Asklipeiou and Kolokotroni Str., 21100 Nafpion, Greece; 12 Intensive Care Unit, "Korgialeneion-Benakeion" Hospital of Athens, 1 Erythrou Stavrou Str., 11526 Athens, Greece; 13 2nd Department of Surgery, University of Thessaloniki, Medical School, 41 Ethnikis Aminis Str., 54635 Thessaloniki, Greece; 14 2nd Department of Internal Medicine, "Sismanogleion" Athens Hospital, 1 Sismanogleiou Str., 15126 Maroussi, Greece; 15 Intensive Care Unit, "Aghia Olga" Athens General Hospital, 3-5 Aghia Olga Str., 14233 Nea Ioania, Greece; 16 Intensive Care Unit, "Thriassio" Elefsina General Hospital, Leoforos Gennimata, 19600 Magoula, Greece; 17 3rd Department of Pulmonary Medicine, "Sismanoglion" Athens Hospital, 1 Sismanogleiou Str., 15126 Maroussi, Greece; 18 5th Department of Internal Medicine, "Evangelismos" Athens Hospital, 45-47 Ispilantou Str., 10676 Athens, Greece; 19 1st Department Propedeutic Surgery, University of Athens, Medical School, 114 Vassilis Sofias Str., 11527 Athens, Greece

## Abstract

**Introduction:**

Although major changes of the immune system have been described in sepsis, it has never been studied whether these may differ in relation to the type of underlying infection or not. This was studied for the first time.

**Methods:**

The statuses of the innate and adaptive immune systems were prospectively compared in 505 patients. Whole blood was sampled within less than 24 hours of advent of sepsis; white blood cells were stained with monoclonal antibodies and analyzed though a flow cytometer.

**Results:**

Expression of HLA-DR was significantly decreased among patients with severe sepsis/shock due to acute pyelonephritis and intraabdominal infections compared with sepsis. The rate of apoptosis of natural killer (NK) cells differed significantly among patients with severe sepsis/shock due to ventilator-associated pneumonia (VAP) and hospital-acquired pneumonia (HAP) compared with sepsis. The rate of apoptosis of NKT cells differed significantly among patients with severe sepsis/shock due to acute pyelonephritis, primary bacteremia and VAP/HAP compared with sepsis. Regarding adaptive immunity, absolute counts of CD4-lymphocytes were significantly decreased among patients with severe sepsis/shock due to community-acquired pneumonia (CAP) and intraabdominal infections compared with sepsis. Absolute counts of B-lymphocytes were significantly decreased among patients with severe sepsis/shock due to CAP compared with sepsis.

**Conclusions:**

Major differences of the early statuses of the innate and adaptive immune systems exist between sepsis and severe sepsis/shock in relation to the underlying type of infection. These results may have a major impact on therapeutics.

## Introduction

The incidence of sepsis has dramatically increased over the past decade. It is estimated that 1.5 million people in the USA and another 1.5 million people in Europe present annually with severe sepsis and/or septic shock: 35 to 50% of them die. The enormous case-fatality had led to an intense research effort to understand the complex pathogenesis of sepsis and to apply the acquired knowledge in therapeutic interventions of immunomodulation [1]. The majority of trials of application of immunomodulatory therapies have failed to disclose clinical benefit probably as a result of the incomplete understanding of the mechanisms of pathogenesis [2]. Populations of patients enrolled in these trials were heterogeneous regarding the type of underlying infection.

Sepsis is accompanied by considerable derangements of both the innate and adaptive immune systems. Changes such as apoptosis of CD4-lymphocytes and of B-lymphocytes and immunoparalysis of monocytes are well recognized among septic patients [3-6]. However, all studies performed so far consider all septic patients to have similar changes of their immune response irrespective of the type of infection that stimulated the septic reaction. If the immune response between septic patients differs in relation to the underlying infection, then many of the disappointing results of clinical trials of immunomodulation may be explained.

The present study was a prospective study undertaken by departments participating in the Hellenic Sepsis Study Group [7]. The aim of the study was to identify if the early statuses of the innate and adaptive immune systems of septic patients differ in relation to the underlying type of infection stimulating the septic response.

## Materials and methods

### Study design

This prospective multicenter study was conducted in 18 hospital departments across Greece between January 2007 and January 2008. Participating departments were: seven ICUs; six departments of internal medicine; one department of pulmonary medicine; three departments of surgery; and one department of urology. A total of 505 patients were enrolled. Written informed consent was provided by the patients or their first-degree relatives for patients unable to consent. The study protocol was approved by the Ethics Committees of the hospitals of the participating centers. Every patient was enrolled once in the study. Patients admitted to the emergency departments, hospitalized in the general ward or the ICU were eligible for the study.

Inclusion criteria were: a) age above 18 years old; b) diagnosis of sepsis, severe sepsis or septic shock; c) sepsis due to either acute pyelonephritis, lower respiratory tract infections, intraabdominal infection, or primary bacteremia; and d) blood sampling within less than 24 hours from the advent of signs of sepsis. The latter inclusion criterion for patients admitted in the emergency departments was defined by their case-history.

Exclusion criteria were: a) HIV infection; b) neutropenia defined as an absolute neutrophil count lower than 1,000 neutrophils/mm^3^; or c) chronic intake of corticosteroids defined as any daily oral intake of 1 mg/kg or more of equivalent prednisone for more than one month. Patients with chronic obstructive pulmonary disease under systemic oral intake of corticosteroids were excluded from the study irrespective of the administered dose regimen.

Sepsis was defined as the presence of a microbiologically documented or clinically diagnosed infection with at least two of the following [8]: a) core temperature above 38°C or below 36°C; b) heart rate of more than 90 beats per minute; c) respiratory rate of more than 20 breaths per minute or partial pressure of carbon dioxide below 32 mmHg; and d) leukocytosis (white blood cell count >12,000/mm^3^) or leukopenia (white blood cell count <4,000/mm^3^) or more than 10% bands in peripheral blood.

Severe sepsis was defined as sepsis aggravated by the acute dysfunction of at least one organ due to tissue hypoperfusion [8]. Septic shock was defined as severe sepsis aggravated by systolic arterial pressure of less than 90 mmHg requiring administration of vasopressors [8].

Acute pyelonephritis was diagnosed in every patient with all the following [9]: a) core temperature above 38°C or below 36°C; b) lumbar tenderness or radiological evidence consistent with the diagnosis of acute pyelonephritis; and c) 10 or more white blood cells per high-power field of spun urine or 2+ or more in dipstick test for white blood cells and nitrates.

Community-acquired pneumonia (CAP) was diagnosed in every patient who was not hospitalized for the past 90 days and who was presenting with all the following [10]: a) core temperature above 38°C or below 36°C; b) white blood cell count of more than 12,000/mm^3^; and c) lobar consolidation in chest x-ray.

Hospital-acquired pneumonia (HAP) was diagnosed in every patient who presented with the following signs at least 48 hours after admission and that were absent upon admission: a) new infiltrate in chest x-ray; and b) clinical pulmonary infection score of 6 or more as defined elsewhere [11].

Ventilator-associated pneumonia (VAP) was diagnosed as HAP presenting in every patient intubated for more than two days with all the following [12-14]: a) core temperature above 38°C or below 36°C; b) purulent tracheobronchial secretions; and c) new chest x-ray infiltrates.

Acute intraabdominal infection was diagnosed in every patient presenting with all the following signs [15]: a) core temperature above 38°C or below 36°C; b) white blood cell count of more than 12,000/mm^3^; and c) radiological evidence on abdominal ultrasound or abdominal computed tomography consistent with the diagnosis of intraabdominal infection.

Primary bacteremia was diagnosed in every patient presenting with all the following [11]: a) peripheral blood culture positive for Gram-positive or Gram-negative bacteria; and b) absence of any alternative site of infection consistent with the pathogen cultured in blood. Isolates of coagulase-negative *Staphylococcus *species or of skin flora isolated from single blood cultures were not considered pathogenic.

Patients were followed-up for 28 days and outcome was recorded. For every patient a complete diagnostic work-out was performed comprising history, thorough physical examination, blood cell counts, blood biochemistry, blood gas, blood culturing from peripheral and central lines, urine cultures, chest x-ray, and chest and abdominal computed tomography or ultrasound if considered necessary. If necessary, quantitative cultures of tracheobronchial secretions or bronchoalveolar lavage were performed and interpreted as already defined [12].

### Blood sampling and laboratory procedure

Within less than 24 hours from the advent of signs of sepsis, 5 ml of blood was sampled by venipuncture of one forearm vein under sterile conditions from every patient. Blood was collected into ethyldiamine tetracetic acid (EDTA)-coated tubes (Vacutainer, Becton Dickinson, Cockeysville, MD, USA) and transported to the central laboratory within less than eight hours at the fourth Department of Internal Medicine at ATTIKON General Hospital of Athens by a courier service for further analysis.

Red blood cells were lysed with ammonium chloride 1.0 mM. White blood cells were washed three times with PBS (pH 7.2) (Merck, Darmstadt, Germany) and subsequently incubated for 15 minutes in the dark with the monoclonal antibodies anti-CD3, anti-CD14 and anti-CD19, and the protein ANNEXIN-V at the fluorochrome fluorescein isothiocyanate (emission 525 nm, Immunotech, Marseille, France); with the monoclonal antibodies anti-CD4, anti-CD8, anti-CD14, anti-CD(16+56) and anti-HLA-DR at the fluorochrome phycoerythrin (emission 575 nm, Immunotech, Marseille, France); with the monoclonal antibody anti-CD3 at the fluorochrome ECD (emission 613 nm, Immunotech, Marseille, France); and with 7-AAD at the fluorochrome PC5 (emission 670 nm, Immunotech, Marseille, France). Fluorospheres (Immunotech, Marseille, France) were used for the determination of absolute counts. The following combinations were applied: anti-CD3/anti-CD4; anti-CD3/anti-CD8; anti-CD3/anti-CD(16+56); ANNEXIN-V/anti-CD4/anti-CD3; ANNEXIN-V/anti-CD8/anti-CD3; ANNEXIN-V/antiCD14; anti-CD14/anti-HLA-DR. Cells were analyzed after running through the EPICS XL/MSL flow cytometer (Beckman Coulter Co, Miami, FL, USA) with gating for mononuclear cells based on their characteristic forward and side scattering. Cells staining negative for CD3 and positive for CD(16+56) were considered as natural-killer (NK) cells. Cells staining positive for both CD3 and CD(16+56) were considered NKT cells. IgG isotypic negative controls at the fluorocolours fluorescein isothiocyanate and phycoerythrin were applied before the start of analysis for every patient. For each cellular subtype, a positive stain for ANNEXIN-V and a negative stain for 7-AAD were considered indicative of apoptosis.

In order to investigate if transportation of EDTA-blood samples may alter the expression of the tested surface antigens, 10 ml of blood were sampled from another nine patients, four with sepsis and five with severe sepsis/shock, all hospitalized in the fourth Department of Internal Medicine at ATTIKON General Hospital of Athens. An aliquot of 5 ml was immediately processed as for any sample. Another 5 ml aliquot was given to the courier service mentioned above for transportation; it was returned to the central laboratory after seven hours. The aliquot was then processed again.

### Statistical analysis

Results were expressed as means ± standard error (SE). As patients with different types of infections differed significantly regarding severity (Table 1) results were expressed separately for patients with sepsis and for patients with severe sepsis/shock. Comparisons of baseline qualitative characteristics were performed by chi-squared test. Comparisons of quantitative variables were performed by analysis of variance (ANOVA) with *post-hoc *Bonferroni adjustment for multiple comparisons to avoid random correlations. Whenever significant differences were disclosed, it was also tested whether these differences were related to final outcome. Results of processing of aliquots immediately after blood sampling and after seven hours of courier transportation were compared by paired t-test. Any value of *P *below 0.05 was considered significant.

**Table 1:** Demographic and clinical characteristics of patients enrolled in the study

	Acute pyeloneprhitis	CAP	Intraabdominal infections	Primary bacteremia	VAP/HAP	*P*
Total number	183	97	100	61	64	
Male/female	86/97	61/36	51/39	41/20	41/23	0.011
Age (years, mean ± SD)	67.3 ± 17.1	68.4 ± 19.7	54.1 ± 24.5	64.0 ± 16.3	70.6 ± 14.5	<0.0001
APACHE II (mean ± SD)	11.7 ± 6.8	15.7 ± 8.8	12.7 ± 7.7	18.2 ± 7.5	20.0 ± 5.4	<0.0001
White blood cells (/μl, mean ± SD)	15684.3 ± 11481.3	15002.2 ± 7272.8	15595.7 ± 7027.8	13755.9 ± 9551.8	13905.7 ± 8289.2	NS
Sepsis/severe sepsis-shock	141/42	56/41	70/30	23/38	22/42	<0.0001
Death (number, %)	14 (7.7)	30 (30.9)	16 (16.0)	21 (34.4)	22 (34.4)	<0.0001
Pathogen* (number, %)						0.039
*Escherichia coli*	71 (38.7)	-	3 (3.0)	12 (19.7)	0 (0)	
*Pseudomonas aeruginosa*	18 (9.8)	-	3 (3.0)	20 (32.8)	15 (23.5)	
*Klebsiella pneumoniae*	12 (6.6)	-	2 (2.0)	12 (19.7)	0 (0)	
*Acinetobacter baumannii*	3 (1.6)	-	0 (0)	10 (16.3)	14 (21.9)	
Other Gram-negatives	9 (4.9)	-	0 (0)	7 (11.5)	1 (1.6)	
*Enterococcus faecalis*	6 (3.3)			1 (1.6)		
Other Gram(+) cocci	5 (2.7)	7 (7.2)	2 (2.0)	4 (6.5)	0 (0)	
Co-morbidities (number, %)						0.045
Diabetes mellitus type 2	48 (26.2)	19 (19.6)	19 (19.0)	16 (26.2)	14 (21.9)	
Heart failure	23 (12.6)	14 (14.4)	9 (9.0)	13 (21.3)	11 (17.2)	
COPD	15 (8.2)	20 (20.6)	5 (5.0)	8 (13.1)	9 (14.1)	
Chronic renal disease	17 (9.3)	6 (6.2)	3 (3.0)	8 (13.1)	7 (10.9)	

*isolated from blood, urine or quantitative cultures of bronchoalveolar lavage and or tracheobronchial secretions;

APACHE: acute physiology and chronic health evaluation; CAP: community-acquired pneumonia; COPD: chronic obstructive pulmonary disease; HAP: hospital-acquired pneumonia; NS: non-significant; SD: standard deviation; VAP: ventilator-associated pneumonia.

## Results

Demographic and clinical characteristics of patients enrolled in the study are shown in Table 1: 183 patients presented with acute pyelonephritis; 97 with CAP; 100 with intraabdominal infection; 61 with primary bacteremia; and 64 with VAP/HAP. *Streptococcus pneumoniae *was isolated either from blood or sputum of seven patients with CAP. Among 100 patients with intraabdominal infections, 28 were suffering from acute ascending cholangitis, 22 from secondary peritonitis after bowel perforation, 22 from acute appendicitis, 12 from liver abscesses, 10 from acute cholocystitis, and six from acute diverticulitis. Six patients with acute cholangitis and two with liver abscesses had secondary Gram-negative bacteremia (Table 1). When acute physiology and chronic health evaluation (APACHE) II score and co-morbidities were compared separately for patients with sepsis and separately for those with severe sepsis/shock no differences were found between different types of infection.

### Characteristics of innate immunity in relation to the underlying infection

No effect of the courier transportation was found in the nine processed samples (Table 2).

**Table 2:** Results of analysis of monocytes and of subsets of lymphocytes of blood samples of nine patients with sepsis processed before and seven hours after courier transportation

	Before transportation	After transportation	*P*
	Mean ± SE	Mean ± SE	
CD14(+)/HLA-DR (+) (%)	91.1 ± 3.8	90.2 ± 3.7	0.588
ANNEXIN-V(+)/CD14(+)/7-AAD(-) (%)	15.47 ± 3.18	12.86 ± 2.60	0.532
CD3(-)/CD(16+56) (mm^3^)	996.8 ± 302.5	904.3 ± 247.4	0.816
ANNEXIN-V(+)/CD(16+56)(+)/CD3(-)/7-AAD(-) (%)	12.53 ± 4.22	16.75 ± 4.37	0.499
CD3(+)/CD(16+56) (mm^3^)	491.9 ± 93.1	455.1 ± 80.2	0.768
ANNEXIN-V(+)/CD(16+56)(+)/CD3(+)/7-AAD(-) (%)	21.04 ± 6.68	20.37 ± 7.93	0.552
CD3(+)/CD4(+) (mm^3^)	3421.7 ± 606.1	3132.7 ± 570.4	0.733
ANNEXIN-V(+)/CD4(+)/CD3(+)/7-AAD(-) (%)	3.08 ± 0.62	2.80 ± 0.71	0.772
CD3(+)/CD8(+) (mm^3^)	1943.6 ± 259.5	2023.8 ± 281.9	0.837
ANNEXIN-V(+)/CD8(+)/CD3(+)/7-AAD(-) (%)	6.35 ± 1.68	6.73 ± 1.82	0.880
CD19 (mm^3^)	363.3 ± 97.7	398.2 ± 123.8	0.828

**Figure 1:**
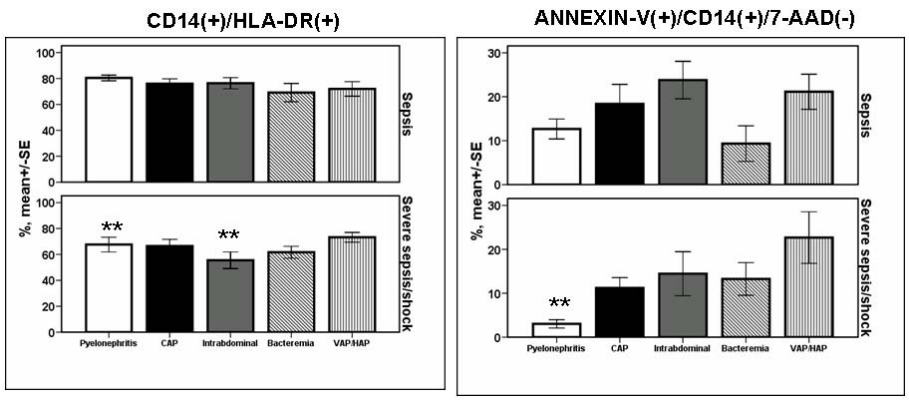
**Expression of HLA-DR on monocytes and rate of apoptosis of monocytes within the first 24 hours of diagnosis among patients with sepsis in relation to the underlying infection**. Patients are divided according to sepsis severity. Double asterisks denote statistically significant differences within the same underlying infection between sepsis and severe sepsis/shock after adjustment for multiple comparisons. CAP: community-acquired pneumonia; HAP: hospital-acquired pneumonia; SE: standard error; VAP: ventilator-associated pneumonia.

Expression of HLA-DR on monocytes and the rate of apoptosis of monocytes did not differ between patients with different types of infection in relation to sepsis severity (Figure 1). However regarding patients with acute pyelonephritis and intraabdominal infection, expression of HLA-DR was significantly decreased among patients with severe sepsis/shock compared with patients with sepsis (*P *of comparisons 0.014 and 0.011, respectively, after adjustment for multiple comparisons). Similar difference was found regarding the rate of apoptosis of monocytes of patients with acute pyelonephritis (*P *< 0.001 after adjustment for multiple comparisons). From the above differences the only one related with final outcome was expression of HLA-DR on monocytes of patients with acute pyelonephritis. Mean ± SE CD14/HLA-DR co-expression of survivors was 79.2 ± 1.99% and of non-survivors 58.2 ± 14.20% (*P *= 0.011 after adjustment for multiple comparisons).

**Figure 2:**
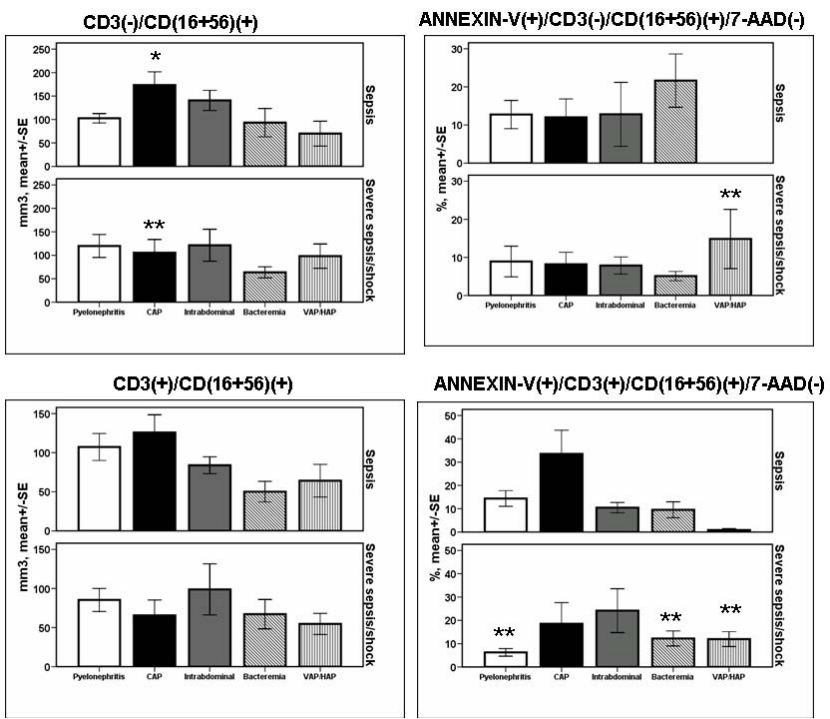
**Absolute counts and rates of apoptosis of NK cells and of NKT lymphocytes within the first 24 hours of diagnosis among patients with sepsis in relation to the underlying infection**. Patients are divided according to sepsis severity. Single asterisk denotes a statistically significant difference between underlying infections after adjustment for multiple comparisons. Double asterisks denote statistically significant differences within the same underlying infection between sepsis and severe sepsis/shock after adjustment for multiple comparisons. CAP: community-acquired pneumonia; HAP: hospital-acquired pneumonia; NK: natural killer; SE: standard error; VAP: ventilator-associated pneumonia.

Regarding patients with sepsis, absolute counts of NK cells were greater among those with CAP compared with the other underlying infections (*P = *0.018 by ANOVA, Figure 2). In patients with VAP/HAP and severe sepsis/shock, the rate of apoptosis of NK cells differed significantly compared with patients with VAP/HAP and sepsis (*P *< 0.001 after adjustment for multiple comparisons). Among patients with acute pyelonephritis or primary bacteremia or VAP/HAP and severe sepsis/shock, the rate of apoptosis of NKT cells differed significantly compared with the rate of apoptosis of patients with similar infections and sepsis (*P *of comparisons 0.035, 0.024 and 0.003, respectively, after adjustment for multiple comparisons).

### Characteristics of adaptive immunity in relation to the underlying infection

**Figure 3:**
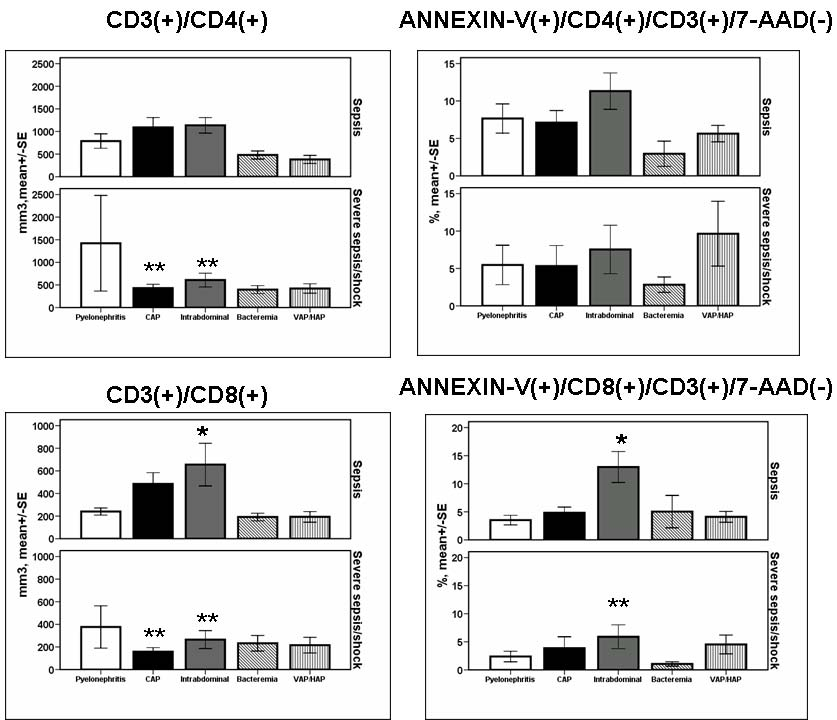
**Absolute counts and rates of apoptosis of CD4- and of CD8-lymphocytes within the first 24 hours of diagnosis among patients with sepsis in relation to the underlying infection**. Patients are divided according to sepsis severity. Single asterisk denotes statistically a significant difference between underlying infections after adjustment for multiple comparisons. Double asterisks denote statistically significant differences within the same underlying infection between sepsis and severe sepsis/shock after adjustment for multiple comparisons. CAP: community-acquired pneumonia; HAP: hospital-acquired pneumonia; SE: standard error; VAP: ventilator-associated pneumonia.

Regarding patients with sepsis, absolute counts of CD8-lymphocytes and their rate of apoptosis were greater among patients suffering from intraabdominal infections compared with patients suffering from other infections (*P *of comparisons 0.008 and 0.001, respectively, by ANOVA, Figure 3). Among patients with CAP or intraabdominal infections and severe sepsis/shock, absolute counts of CD4-lymphocytes were significantly decreased compared with patients with CAP or intraabdominal infections and sepsis (*P *of comparisons 0.024 and 0.027 after adjustment for multiple comparisons). In severe sepsis/shock due to CAP, absolute counts of CD8-lymphocytes were significantly decreased compared with CAP and sepsis (*P *= 0.014 after adjustment for multiple comparisons). The rate of apoptosis of CD8-lymphocytes was significantly decreased among patients with intraabdominal infections and severe sepsis/shock compared with patients with intraabdominal infections and sepsis (*P *= 0.050 after adjustment for multiple comparisons).

Mean ± SE absolute CD4-lymphocyte count of survivors with CAP was 965.4 ± 179.4 mm^3 ^and of non-survivors with CAP 414.3 ± 126.9 mm^3 ^(*P *= 0.019 after adjustment for multiple comparisons). Mean ± SE absolute CD8-lymphocyte count of survivors with CAP was 411.5 ± 83.5 mm^3 ^and of non-survivors with CAP 169.0 ± 47.1 mm^3 ^(*P *= 0.015 after adjustment for multiple comparisons).

**Figure 4:**
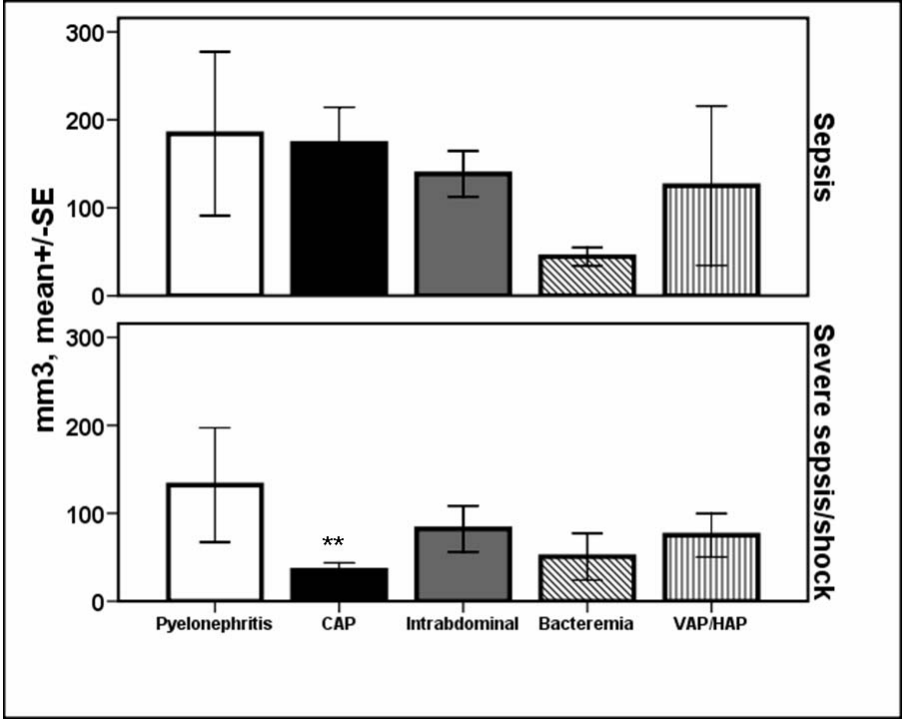
**Absolute counts of B-lymphocytes within the first 24 hours of diagnosis among patients with sepsis in relation to the underlying infection**. Patients are divided according to sepsis severity. Double asterisks denote statistically significant differences within the same underlying infection between sepsis and severe sepsis/shock after adjustment for multiple comparisons. CAP: community-acquired pneumonia; HAP: hospital-acquired pneumonia; SE: standard error; VAP: ventilator-associated pneumonia.

Absolute counts of B-lymphocytes were significantly decreased among patients with CAP and severe sepsis/shock compared with CAP and sepsis (p: 0.003 after adjustment for multiple comparisons; Figure 4). Mean ± SE absolute B-lymphocyte count of survivors with CAP was 137.1 ± 34.2 mm^3 ^and of non-survivors with CAP 56.9 ± 17.1 mm^3 ^(*P *= 0.042 after adjustment for multiple comparisons).

### Characteristics of innate and adaptive immunity in relation to the implicated pathogens

**Figure 5:**
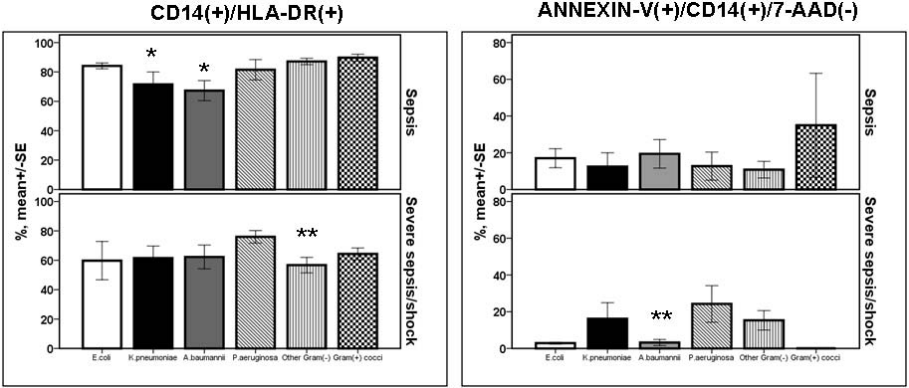
**Expression of HLA-DR on monocytes and rate of apoptosis of monocytes within the first 24 hours of diagnosis among patients with sepsis in relation to the implicated pathogens**. Patients are divided according to sepsis severity. Single asterisk denotes a statistically significant difference between underlying infections after adjustment for multiple comparisons. Double asterisks denote statistically significant differences within the same underlying infection between sepsis and severe sepsis/shock after adjustment for multiple comparisons. SE: standard error.

**Figure 6:**
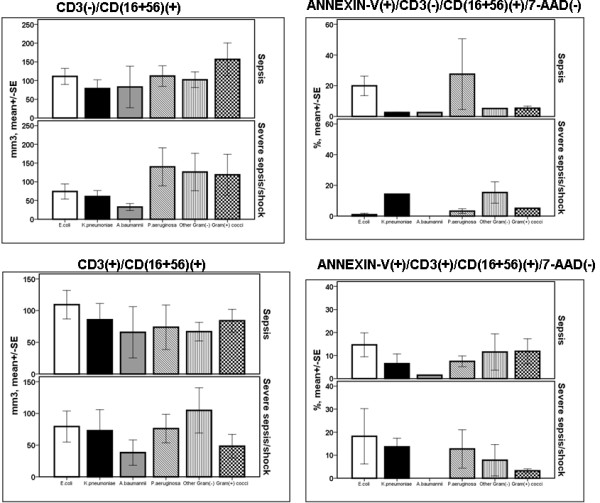
**Absolute counts and rates of apoptosis of NK cells and of NKT lymphocytes within the first 24 hours of diagnosis among patients with sepsis in relation to the implicated pathogens**. Patients are divided according to sepsis severity. NK: natural killer; SE: standard error.

**Figure 7:**
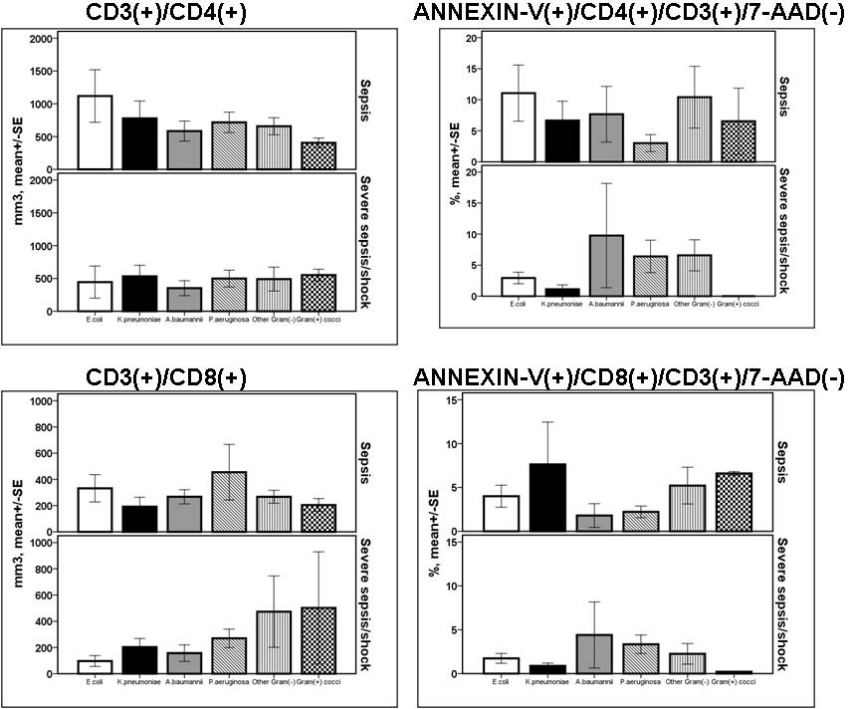
**Absolute counts and rates of apoptosis of CD4- and of CD8-lymphocytes within the first 24 hours of diagnosis among patients with sepsis in relation to the implicated pathogens**. Patients are divided according to sepsis severity. SE: standard error.

**Figure 8:**
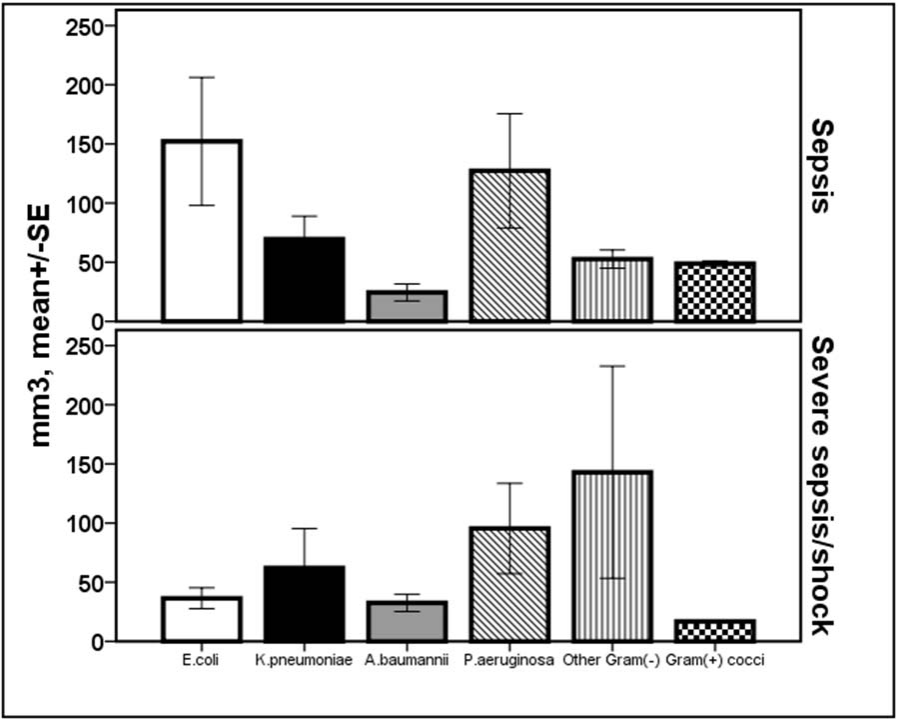
**Absolute counts of B-lymphocytes within the first 24 hours of diagnosis among patients with sepsis in relation to the implicated pathogens**. Patients are divided according to sepsis severity. SE: standard error.

In order to study if the described differences are related to the type of implicated bacterial species, groups of infections by bacterial species are defined. Results are shown in Figures 5, 6, 7 and 8. Regarding patients with sepsis infected by isolates of *Klebsiella pneumoniae *and *Acinetobacter baumannii *expression of HLA-DR on monocytes was lower compared with patients infected by other isolates (*P *= 0.023 by ANOVA). Such differences were not found among patients with severe sepsis/shock (Figure 5). The rate of apoptosis of monocytes was lower among patients infected by *A. baumannii *and severe sepsis/shock compared with patients infected by *A. baumannii *and sepsis (*P *= 0.042 after adjustment for multiple comparisons).

No differences were encountered among patients infected by different bacterial species regarding NK cells, NKT cells, CD4-lymphocytes, CD8-lymphocytes, B-lymphocytes and their rates of apoptosis (Figures 6, 7 and 8).

## Discussion

The great rate of mortality associated with severe sepsis and septic shock has stimulated research to try to understand the complex pathogenesis. Numerous randomized clinical trials have been conducted with the administration of agents modulating the immune response of the host. Results of these trials were controversial. It has been hypothesized that part of this controversy is due to the enrolment of heterogeneous patient populations [2]. Pathogenesis of sepsis has been studied under the assumption that all types of infection may stimulate a similar inflammatory reaction.

No study similar in design to the current study has been published, at least to our knowledge, to try to compare the early innate and adaptive immune responses of septic patients with different types of infection. Early innate immune response of the septic host comprises recognition of well-conserved structures of the offending pathogens, known as pathogen-associated molecular patterns (PAMPs), by pattern recognition receptors (PRRs) located either in the cell membrane or inside the cytoplasm of blood monocytes and tissue macrophages. Endotoxins of the cell wall of Gram-negative bacteria and peptidoglycan of the cell wall of Gram-positive cocci are among the best studied PAMPs. The best studied PRRs are toll-like receptors (TLRs) that are transmembrane receptors of blood monocytes and tissue macrophages; once stimulated by their agonists they produce pro-inflammatory cytokines [15]. When monocytes of the septic host are stimulated *ex vivo *they fail to produce a similar amount of cytokines as monocytes of the non-septic host. This phenomenon is called immunoparalysis and it may be accompanied by cellular apoptosis. In a recent study by our group, monocytes were isolated from 36 patients with sepsis due to VAP and compared with 32 patients with sepsis caused by other types of infections. Patients were well matched for disease severity. The rate of apoptosis of monocytes was greater among patients with sepsis due to VAP than sepsis of other etiology. Among patients with VAP, immunoparalysis of monocytes was linked with unfavorable outcome, which was not found among patients with sepsis of other etiology [16]. Expression of TLRs was not assessed in the present study. Instead activation of monocytes was assessed by the expression of HLA-DR on the cell surface; decrease of CD14/HLA-DR co-expression is considered an index of immunoparalysis and bad prognosis [17]. The latter decrease was only shown for patients with severe sepsis/shock due to acute pyelonephritis and acute intraabdominal infections (Figure 1).

Sequential results of both animal and human studies favor a detrimental role for NK cells in sepsis. Murine models of pneumococcal pneumonia [18], multiple trauma [19] and abdominal sepsis [20,21] reveal that depletion of NK cells prolongs survival and attenuates the systemic inflammatory reaction whereas the presence of NK cells is consistent with amplification of the inflammatory reaction. This is indirectly shown in humans after measurement of serum concentrations of granzymes A and B that are released after activation of NK cells. Concentrations of granzymes A and B are increased in healthy volunteers subject to experimental endotoxemia and in patients with melioidosis and bacteremia [22].

Increase of the absolute counts of NK cells was a profound change of sepsis due to CAP. The rate of apoptosis of NK cells and of NKT cells was more increased among patients with VAP/HAP and severe sepsis/shock than among those with VAP/HAP and sepsis. That was also the case for the rate of apoptosis of NKT cells among patients with primary bacteremia, whereas the opposite was found regarding the rate of apoptosis of NKT cells among patients with acute pyelonephritis (Figure 2). Whether the increase of NK cells in CAP is related to the underlying microbiology of patients is not known. The exact microbiology was not known for all of these patients (Table 1). As *S. pneumoniae *is the main causative pathogen of CAP, it may be hypothesized that the greater absolute counts of NK cells in that study population may be related to the different stimulation of the immune system by Gram-positive cocci and by Gram-negative bacteria. Thorough analysis of data of the present study failed to document the existence of such differences (Figures 5, 6, 7 and 8). A link between the type of bacterial pathogens and subsets of lymphocytes in sepsis has been shown in a study enrolling a limited number of patients. More precisely, 10 patients with Gram-positive sepsis were compared with 10 patients with Gram-negative sepsis. Absolute counts of NK cells, CD4-lymphocytes and CD8-lymphocytes were estimated. No differences were found within the first 24 hours; however, NK cell count was greater among patients with sepsis of Gram-positive origin than among patients with Gram-negative sepsis on days 7 and 14 [23].

Regarding early changes of the adaptive immunity, it was found that the absolute counts of CD8-lymphocytes are particularly elevated among patients with sepsis due to intraabdominal infections than other types of infection. A decrease of CD4-lymphocytes of patients with CAP or intraabdominal infections and severe sepsis/shock was found compared with CAP or intraabdominal infections and sepsis. The rate of apoptosis of CD8-lymphocytes was also decreased among patients with intraabdominal infections and severe sepsis/shock compared with patients with intraabdominal infections and sepsis.

Early T-lymphopenia occurs in sepsis due to the migration of cells from the systemic circulation to the infection site [24,25]. Several studies of experimental sepsis in mice have shown that CD4-lymphocytes play a pivotal role in the attempt to withhold infection spread and to format an abscess [25-27]. CD4-lymphocyte counts have also been described to be lower among patients with sepsis due to VAP than among patients with sepsis due to other types of infection [16].

Early changes of the adaptive immune system also involved B-lymphocytes. They were decreased among patients with CAP and severe sepsis/shock compared with patients with CAP and sepsis.

Main limitations of the present study are: a) the lack of information about the expression of TLRs on blood monocytes; b) limited information about the microbiology of patients with CAP; c) lack of information about the kinetics of subsets of lymphocytes over follow-up of the enrolled patients; and d) the smaller number of enrolled patients with primary bacteremia and VAP/HAP compared with the other types of infections that may not allow for some differences in cell populations to be shown. Despite these limitations, it may be hypothesized that early statuses of the innate and of the adaptive immune systems during transition from sepsis to severe sepsis/shock differ according to the underlying type of infection. In the field of acute pyelonephritis expression of HLA-DR on monocytes, the rate of apoptosis of monocytes and the rate of apoptosis of NKT cells decrease; in CAP absolute counts of NK cells, CD4-lymphocytes, CD8-lymphocytes and B-lymphocytes decrease; in intraabdominal infections absolute counts of CD8-lymphocytes and the rate of apoptosis of CD8-lymphocytes decrease; in primary bacteremia the rate of apoptosis of NKT cells increase; and in VAP/HAP the rate of apoptosis of NKT cells and of NK cells increase. However, factors such as prolonged stay in the ICU and co-morbidities may also play some role in these differences. The bacterial origin of sepsis does not seem to be involved in these differences. The great majority of isolated pathogens were Gram-negatives and no connection was found between the bacterial origin of sepsis and the estimated parameters (Figures 5, 6, 7 and 8).

## Conclusions

The presented results reveal that major differences of the early statuses of the innate and adaptive immune systems exist between sepsis and severe sepsis/shock in relation to the underlying type of infection. These results may have a major impact on therapeutics so that the strategy of therapeutic immunointervention may be directed by the type of underlying infection.

## Key messages

• Early statuses of the innate and adaptive immune system in patients with sepsis differ according to the underlying type of infection.

• These differences are particularly found on transition from sepsis to severe sepsis/shock.

## Abbreviations

ANOVA: analysis of variance; APACHE: acute physiology and chronic health evaluation; CAP: community-acquired pneumonia; EDTA: ethyldiamine tetracetic acid; HAP: hospital-acquired pneumonia; NK: natural killer; PAMPs: pathogen-associated molecular patterns; PBS: phosphate buffered saline; PRRs: pattern recognition receptors; SE: standard error; TLR: toll-like receptor; VAP: ventilator-associated pneumonia.

## Authors' contributions

CG analyzed data and drafted the manuscript. AA, AP, GG, IV, PM, VK, HK, AP, and AS performed the experiments. SA, ZA, GA, AA, SA, FB, AC, SC, ID, IF, EG, PG, AI, KK, NK, IK, GK, PK, IK, PK, ML, KL, KL, EM, AM, CM, IM, MM, MM, HM, VM, IP, KP, MP, IP, DP, KP, KR, VS, MS, VS, MS, IS, and TT collected clinical data and blood samples. KM, PKM, NM, HG and AA participated in study design and drafted the manuscript. EJGB designed the study and wrote the manuscript.

## Competing interests

The authors declare that they have no competing interests.
